# Treatment of Ammonia Nitrogen Wastewater in Low Concentration by Two-Stage Ozonization

**DOI:** 10.3390/ijerph120911975

**Published:** 2015-09-23

**Authors:** Xianping Luo, Qun Yan, Chunying Wang, Caigui Luo, Nana Zhou, Chensheng Jian

**Affiliations:** 1 School of Resuorces and Environmental Engieering, Jiangxi University of Science and Technology, Ganzhou 341000, Jiangxi, China; E-Mails: yanqun8219893@163.com (Q.Y.); cywang@jxust.edu.cn (C.W.); andk24@163.com (C.L.); hdongxue@163.com (N.Z.); gctao@163.com (C.J.); 2 Western Mining Co., Ltd., Xining 810006, Qinghai, China; 3 Jiangxi Key Laboratory of Mining & Metallurgy Environmental Pollution Control, Jiangxi University of Science and Technology, Ganzhou 341000, Jiangxi, China

**Keywords:** ammonia nitrogen wastewater, ozone oxidation, mechanism

## Abstract

Ammonia nitrogen wastewater (about 100 mg/L) was treated by two-stage ozone oxidation method. The effects of ozone flow rate and initial pH on ammonia removal were studied, and the mechanism of ammonia nitrogen removal by ozone oxidation was discussed. After the primary stage of ozone oxidation, the ammonia removal efficiency reached 59.32% and pH decreased to 6.63 under conditions of 1 L/min ozone flow rate and initial pH 11. Then, the removal efficiency could be over 85% (the left ammonia concentration was lower than 15 mg/L) after the second stage, which means the wastewater could have met the national discharge standards of China. Besides, the mechanism of ammonia removal by ozone oxidation was proposed by detecting the products of the oxidation: ozone oxidation directly and ·OH oxidation; ammonia was mainly transformed into NO_3_^−^-N, less into NO_2_^−^-N, not into N_2_.

## 1. Introduction

Wastewater generated during rare earth production is radioactive, ammonia-containing, fluoride-containing, acid or alkaline [1]. In recent years, the long-term mining of rare earth ore in southern Jiangxi, China, produced large amounts of ammonia nitrogen wastewater in low concentrations. The wastewater that is rich in ammonia nitrogen would inhibit the natural nitrification, cause water hypoxia, result in fish poisoning, decrease the water purification capacity, and finally do great harm to the water environment [2]. NH_3_, as a neutral molecule, is able to diffuse across the epithelial membranes of aquatic organisms much more readily than the charged ammonia ion. It was reported that, ammonia could block oxygen transfer in the gills of fish. Fish suffering from ammonia poisoning appear sluggish, and come to the surface of water gasping for air. In marine environments, the safe level of ammonia is below 1 mg/L [3]. In China, the primary standard of ammonia nitrogen in wastewater is less than 15 mg/L and the secondary standard is less than 50 mg/L (Integrated wastewater discharge standard (GB 8978-1996)). The methods of chemical precipitation, blow-off, and adsorption are commonly used for the treatment of ammonia nitrogen wastewater at low concentration. Chemical precipitation method intends to reduce the water solubility of ammonia nitrogen by the formation of indissoluble salt; blow-off method is typically used NaOH to adjust pH to basic of wastewater and ammonia nitrogen would exist in the form of free ammonia (NH_3_). Then, ammonia nitrogen would escape from aqueous solution to the atmosphere. Besides, Biological nitrification as the most reliable method for the removal of ammonia has been established widely. However, biological nitrification beds are subject to great fluctuations in efficiency, as nitrifying bacteria in biofilter beds are sensitive to environmental perturbations and changes in operating conditions, which often implicate color, odor and flavor problems and moreover impair biofilter function [4]. All methods have their own characteristics, but each has its limitations, or has different levels of equipment investment, high operating costs, secondary pollution, and other shortcomings [5,6,7,8,9]. Therefore, it is necessary and urgent to seek efficient, practical, low concentration ammonia removal methods.

Ozone is a strong oxidizer, especially when there coexist a lot of OH^−^, H_2_O_2_/HO^−^, Fe^2+^, UV and other free radical activators or accelerators [10,11]. Under the action of excited agent radicals and the accelerator, the ozone would induce large amounts of hydroxyl radicals (·OH) in the reaction system, which would lead to chain reactions and then produce more reactive radicals. Besides, ozone is easy to operate. So, ozone treatment is one of the most common processes utilized in industry: decolorization of wastewater containing direct dye (Sirius Blue SBRR) by ozonization was studied in an attempt to abate pollution caused by textile dyeing houses and dye-producing plants [12]; domeno reported that ozonization was one of the most efficient treatment of volatile organic compounds and odors from gaseous emissions [13]; ozone could be used to remove metal Tin from ITO-scrap [14]. Thus, ozone oxidation technology is favored by researchers in the treatment of various pollutants. Ammonia nitrogen was also treated by ozonization [15,16,17]. As other oxidants [6,18], ozone could convert highly toxic ammonia and nitrite to nitrate with low toxicity [15].

In order to meet the discharge standard of China, the ammonia nitrogen wastewater at a medium or low concentration of about 100 mg/L was treated by two-stage ozone oxidation method in this study. Effects of ozone flow rate, initial pH of reaction solution, and reaction time on the ammonia nitrogen removal were investigated by ozone wet oxidation. The transformation process of ammonia nitrogen was analyzed to deduce the mechanism of ammonia nitrogen removal.

## 2. Experimental Section

### 2.1. Experimental Reagents and Apparatus

**Figure 1:**
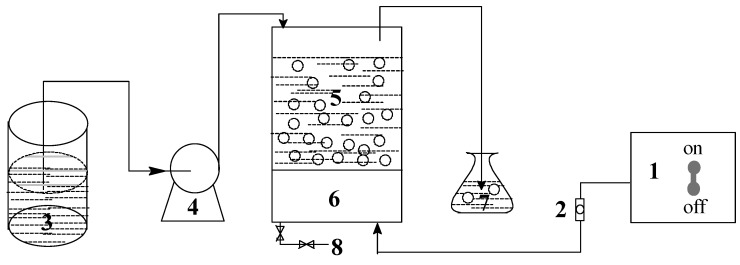
The flowchart of experimental apparatus. (**1**) Ozone generator; (**2**) Gas flowmeter; (**3**) Raw water tank; (**4**) Constant flow pump; (**5**) Reaction column; (**6**) Air distribution plate; (**7**) Tail gas absorption bottle; (**8**) Outlet.

Ammonium chloride, mercuric iodide, potassium persulfate, and hydrochloric acid were of analytical grade and used without any further purification; ozone was provided by LH-YT-10G ozone generator (Tongfang Environment Co., Ltd., Beijing, China); pH and absorbance values were determined by REX PHS-3C pH meter (Shanghai INESA Scientific Instrument Co., Ltd., Shanghai, China) and SP-1920 UV spectrophotometer (Unico (Shanghai) Instrument Co., Ltd., Shanghai, China) respectively. The experimental apparatus is a cylindrical reactor made of transparent glass, and the oxidation system comprises several operation units as shown in Figure 1.

### 2.2. Analysis Method

Ammonia-containing wastewater (100 ± 10 mg/L, pH 6–6.2) was prepared by ammonia chloride. At room temperature (about 25 °C), 500 mL of water sample was added into the reactor after the adjustment of pH, then ozone was pumped in. The reaction solution was sampled every 20 min to analyze the changes of pH values, concentrations of ammonia nitrogen (NH_4_^+^-N), nitrite nitrogen (NO_2_^−^-N), nitrate nitrogen (NO_3_^−^-N), and total nitrogen (TN). pH value was measured by pH meter, and the concentrations of chemicals above were measured by spectrophotometry methods as shown in Table 1. Ozone flow rate, reaction time, and pH values were the main effects considered in the experiment of ammonia removal by ozone wet oxidation. The removal efficiency (*R*) was calculated by Equation (1) as follows:

(1) $$R=(C_{0}-C)/C_{0}\times 100\%$$

where *C*_0_ is the initial concentration of ammonia nitrogen and *C* is the concentration at reaction time *t* (min).

**Table 1:** Methods of determination.

Project Name	Determination	Standard Number	Reference
Ammonia nitrogen	Nessler’s reagent spectrophotometry	HJ 535-2009	[19]
Nitrite	N-(1-naphthyl)-ethylenediamine spectrophotometry	GB 7493-87	[20]
Nitrate	Phenol disulfonic acid spectrophotometry	GB 7480-87	[21]
Total nitrogen	Alkaline potassium persulfate digestion spectrophotometry	HJ 636-2012	[22]

There are two processes of ammonia removal by ozone wet oxidation including oxidation by ozone and ammonia nitrogen stripping by ozone flow. An air stripping experiment was set as a blank experiment to simulate the stripping removal of ammonia by ozone.

## 3. Results and Discussion

### 3.1. Blank Stripping

**Figure 2:**
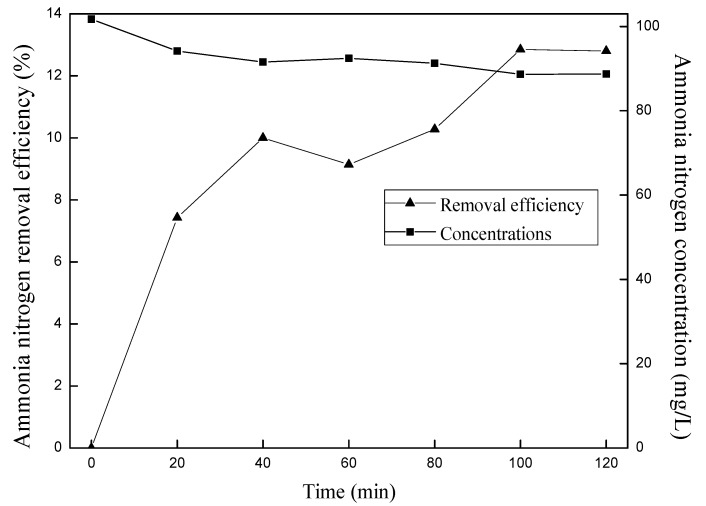
Curves of blank stripping over 3L/min air stream.

Air flow rate in the stripping experiment was over 3 L/min and the pH of NH_4_^+^-N solution was adjusted to 11. The curves of ammonia concentration and ammonia removal efficiency *versus* stripping time were seen in Figure 2. Ammonia nitrogen removal efficiency increased with the prolonging of time until 100 min, and there was no significant change to 120 min. In addition, the maximum removal efficiency was only 12.8% by air stripping with the flow rate over 3 L/min. Ammonia removal efficiency in ozone oxidation system should be smaller than 12.8% because ozone flow rate would be set smaller than 3 L/min.

### 3.2. Effects of Ozone Concentrations on Ammonia Removal

First, the outlet of ozone generator was inserted directly into an exhaust absorption flask containing potassium iodide solution. Then, the ozone was transferred into another exhaust gas absorption flask as same as the first one when the ozone pressure, current, and flow rate were stable. After a certain period, the power was turned off and the residual potassium iodide was titrated by the sodium thiosulfate standard solution [23]. The concentration of ozone was calculated by Equation (2), and the relationship between the ozone concentration and the flow rate is shown in Table 2.

(2) *C*o_3_ = *A*_Na_ × *B* × 24.00/*V*_0_ (mg/L)

where *C*o_3_ is ozone concentration, mg/L; *A*_Na_ is the consumption of sodium thiosulfate standard solution, mL; *B* is the concentration of sodium thiosulfate standard solution, mol/L; *V*_0_ is the volume of ozone gas, L.

**Table 2:** The relationship between ozone concentration and flow rate.

**Ozone Flow Rate (L/min)**	0	0.3	0.5	0.8	1.0	1.3
**Ozone Concentration (mg/L)**	0	25.83	30.24	51	58.14	71.82

**Figure 3:**
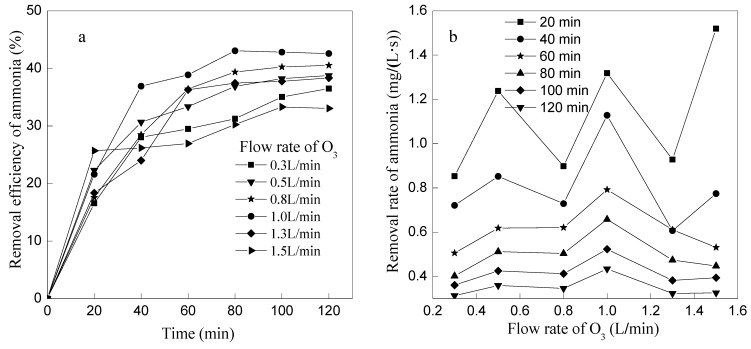
Effect of ozone flow rate on ammonia removal. (**a**) The effect of ozone flow rate on removal efficiency of ammonia; (**b**) The effect of ozone flow rate on removal rate of ammonia.

Experiments were performed with pH 10 of the initial solution in the system at different ozone flow rates (0.3, 0.5, 0.8, 1.0, 1.3 and 1.5 L/min). The effects of different ozone flow rates on the ammonia removal efficiency and ammonia removal rate are shown in Figure 3.

As seen from Figure 3a, the removal efficiency of ammonia nitrogen all increased gradually *versus* time at different ozone flow rates till 100 min. The removal efficiency increased with the increase of ozone flow rate when the ozone flow rate was less than 1 L/min. However, the ammonia removal efficiency decreased with the increase of ozone flow rate when ozone flow rate was more than 1 L/min. So, the optimum ozone flow rate of ammonia removal by ozone oxidation was 1 L/min in this study. From another point of view, as seen in Figure 3b, the ammonia removal rate decreased with the increase of ozone flow rate when ozone flow rate ≥1 L/min. The reason for this phenomenon may be that greater ozone flow could not support enough time for the contact between ozone molecules and OH^−^, which would lead to the slow generation rate of ·OH, as showed in Equations (3) and (4); besides, the excessive flow would go against the direct ammonia oxidation by ozone. Ammonia removal efficiency reached 43.06% and pH reduced to 7.1 under the conditions of 1 L/min ozone flow rate and 80 min reaction time.

(3) O_3_ + OH^−^ → HO_2_^−^ + O_2_

(4) O_3_ + HO_2_^−^ → ·OH + O_2_^−^ + O_2_

**Figure 4:**
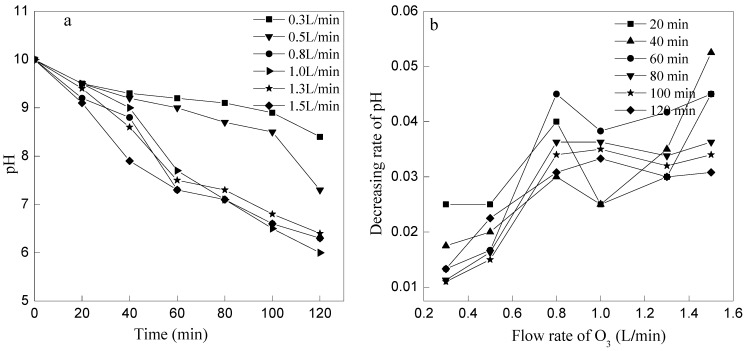
Variation of pH *versus* reaction time at different ozone flow rates. (**a**) The changes of pH at different ozone flow rate; (**b**) The decreasing rate of pH at different ozone flow rate.

pH value of wastewater decreased gradually with the increasing of the removal reaction (Figure 4a) and the decreased rate of pH increased first and then decreased with the ozone flow increased (Figure 4b). The reason of pH value decreased was mainly due to the continuous production of H^+^ during the oxidation process of ammonia and the consumption of OH^−^ by O_3_. There was no big difference to the changes of pH value among 0.8, 1, 1.3, and 1.5 L/min of ozone flow rate, but there was the lowest pH value when ozone flow rate was 1.0 L/min after 120 min. As mentioned above, OH^−^ was induced to ·OH in the process of ozone wet oxidation, and it was unfavorable for the ·OH production if the ozone flow rate was too fast or too slow. Therein, the ammonia removal efficiency and the production H^+^ were all decreased when the ozone flow rate was less or more than 1.0 L/min.

### 3.3. Effect of Initial pH on Ammonia Removal by Ozone Oxidation

**Figure 5:**
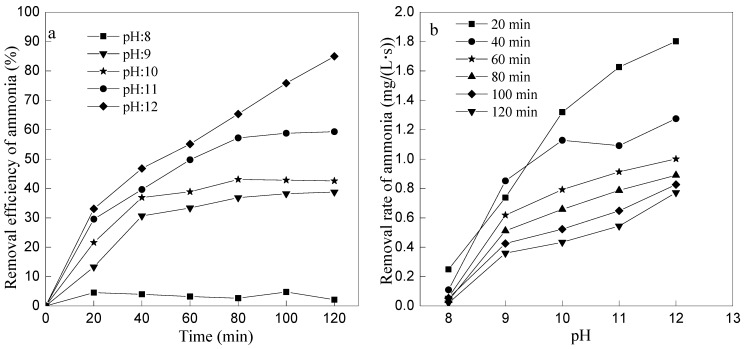
Effect of initial pH on oxidation of ammonia. (**a**) The effect of initial pH on removal efficiency of ammonia; (**b**) The effect of initial pH on removal rate of ammonia.

**Figure 6:**
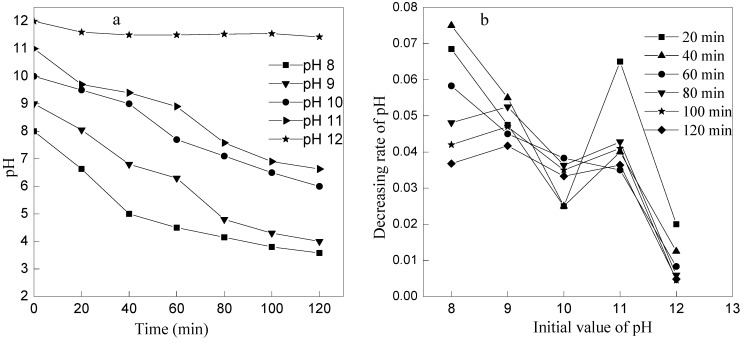
Variation of pH with reaction time at different initial pH. (**a**) The changes of pH at different initial pH; (**b**) The decreasing rate of pH at different initial pH.

To investigate the effect of initial pH on ammonia removal by ozone oxidation, a series of experiments were designed with different initial pH values (8, 9, 10, 11, and 12) at the ozone flow rate of 1 L/min. The result is shown in Figure 5 and Figure 6.

The initial pH value of the reaction solution has important effect on the ammonia removal by ozone oxidation. The higher of the pH value, the larger of the ammonia nitrogen removal efficiency. There was almost no removal of ammonia at initial pH 8 while the removal efficiency reached 84.97% at initial pH 12 after 120 min.

It is observed from Figure 6 that pH value of the wastewater kept reducing with the reaction time prolonged, and, the smaller of initial pH value, the greater of changing pH rate. However, the pH decreased slowly when initial pH was 12, which reduced to pH 11.43 at the reaction time of 120 min. If the initial pH was adjusted to 12, it would require large amount of acid to neutralize the excessive alkalis (pH value is 11.43) in order to meet the discharge standard. The efficiency of ammonia removal reached 57.20% and pH value decreased to 7.58 after 80 min at initial pH 11, which was not only higher than the efficiency at initial pH 10 by14%, but also consumed much less acid than that at an initial pH value of 12. So, pH value is 11 was chose to be the optimum initial pH value at ozone flow rate 1 L/min.

### 3.4. Mechanism Analysis

There are two forms of ammonia nitrogen in water solution, free ammonia (NH_3_) and ammonium ion (NH_4_^+^), which are reversible, as seen in Equation (5). The composition ratio of NH_3_ to NH_4_^+^ depends on the pH value of water and the water temperature. The higher pH value, the higher proportion of NH_3_; conversely, the ammonium ion proportion is higher at a lower pH value. At pH 7 of the waste water, ammonia nitrogen exists mainly in the form of NH_4_^+^, while more than 90% [24] is free ammonia when the pH is over 11. In addition, during the ozone oxidation process of ammonia, the reaction rate constant of molecular ozone and ammonia was 20 L·(mol·s)^−1^, and the reaction rate constant of molecular ozone and ammonium ion is only 1 L·(mol·s)^−1^ [25]. The capacity of oxidative degradation of O_3_ on ammonia nitrogen increases with the improvement of the pH value. Besides, high pH could accelerate the decomposition of O_3_, and then induce the generation of ·OH, which has strong oxidative ability. So, the degradation of ammonia nitrogen by ozone wet oxidation includes both O_3_ molecular oxidation directly (Equations (6)–(10)) and ·OH oxidation reaction (Equations (3), (4), and (11)–(13)) [26]. Ozone oxidation predominates the reaction at low pH, while ·OH oxidation is the main reaction at high pH, and ·OH oxidation has the faster reaction rate [27,28]. As discussed in Section 3.2 and Section 3.3 of this study, the ozone flow rate would affect the contacting time of reaction between ozone molecules or ·OH and free ammonia, so ozone flow rate of 1 L/min and initial pH of 11 were chosen as the optimum conditions in the process of ammonia nitrogen removal by ozone oxidation.

(5) NH_4_^+^ + OH^−^ ⇋ NH_3_ + H_2_O

(6) 3O_3_ + NH_4_^+^ → NO_2_^−^ + 2H^+^ + H_2_O + 3O_2_

(7) NO_2_^−^ + O_3_ → NO_3_^−^ + O_2_

(8) 4O_3_ + NH_4_^+^ → NO_3_^−^ + 2H^+^ + H_2_O + 4O_2_

(9) 3O_3_ + NH_3_ → NO_2_^−^ + H^+^ + H_2_O + 3O_2_

(10) 4O_3_ + 3NH_3_ → NO_3_^−^ + H^+^ + H_2_O + 4O_2_

(11) 6·OH + NH_3_ → NO_2_^−^ + H^+^ + 4H_2_O

(12) NO_2_^−^ + 2·OH → NO_3_^−^ + H_2_O

(13) NH_3_ + 8·OH → NO_3_^−^ + H^+^ + 5H_2_O

The products of ammonia nitrogen degradation could be NO_3_^−^ and NO_2_^−^ by above equations. In order to confirm the final product of ammonia nitrogen oxidation by ozone, concentrations of NO_3_^−^, NO_2_^−^ and TN were measured at the ozone flow rate of 1 L/min and pH 10, pH 11, respectively.

**Figure 7:**
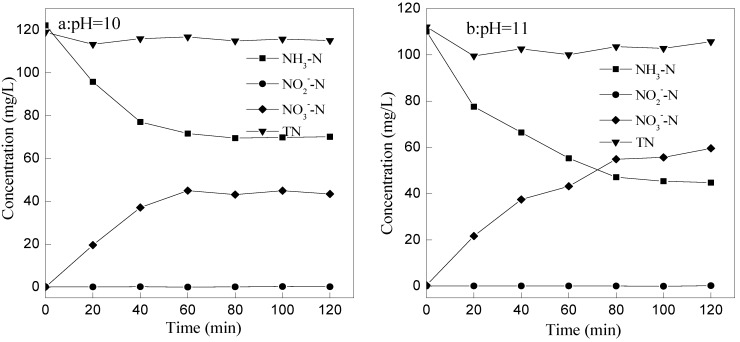
The concentration changes of NH_4_^+^-N, TN, NO_3_-N, and NO^2^^−^-N with time. (**a**) At pH 10; (**b**) At pH 11.

As seen from Figure 7, content of NO_3_^−^-N increases with the degradation of ammonia nitrogen, and the NO_3_^−^-N concentration also tends to be smooth while the concentration of ammonia nitrogen tends to be stable. Concentration of nitrite had been very low, less than 1 mg/L, due to the instability of nitrite, which is easily oxidized to nitrate.

At the beginning of reaction, TN decreased slightly. After 40 min of the reaction, TN increased slightly, and this might be due to the external nitrogen in the atmosphere. Therefore, the TN content had little change through the whole process, which means that the ammonia removal effect of stripping is less than the oxidation of ozone. In another word, NH_4_^+^-N, NO_3_-N, NO_2_-N, and TN are in dynamic equilibrium, and ammonia nitrogen is difficult to convert to N_2_ in the ozone oxidation process.

## 4. The Second Stage Treatment of Ammonia Nitrogen Wastewater

The ammonia removal efficiency reaches 59.32% by the primary treatment of ozone oxidation at the conditions of 1 L/min ozone flow rate, initial pH 11, and 120 min of the reaction. However, it still does not meet the primary discharge standard of China. Therefore, two-stage treatment of ammonia nitrogen wastewater was introduced: the wastewater treated by the primary treatment was treated again by ozone wet oxidation with the same ozone flow rate of 1 L/min, and pH value was regulated to 11 again.

**Figure 8:**
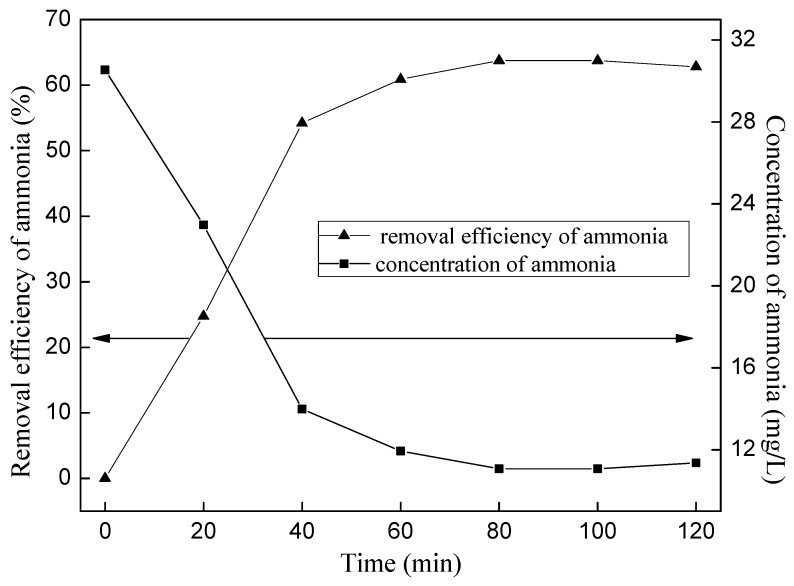
The removal of ammonia by two stages of ozone oxidation.

After being treated by the second stage of ozone oxidation, the ammonia removal efficiency reached more than 85% and ammonia concentration was below 15 mg/L (Figure 8), which meets the national discharge standards of China.

## 5. Conclusions

By the two-stage treatment of ozone wet oxidation, ammonia nitrogen wastewater with the original concentration of 100 ± 10 mg/L could meet the national discharge standard of China. The detailed following conclusions can be drawn as follows:

(1) Ammonia removal efficiency increased with the ozone flow rate increased when ozone flow rate was ≤1 L/min, while the ammonia removal efficiency decreased with increasing of ozone flow rate when ozone flow rate was ≥1 L/min; the higher of the initial pH, the higher of the ammonia nitrogen removal efficiency. However, too much acid would be needed to neutralize the excessive alkalis if the initial pH > 11; at the optimum conditions of 1 L/min ozone flow rate and initial pH 11, ammonia removal efficiency could reach 59.32% and pH reduced to 6.63 after 120 min by the primary ozone oxidation, and the efficiency could reach 85% after the second stage of ozone oxidation, which would meet the discharge standard of China.
(2) During the ozonization of ammonia nitrogen, there is a certain contribution to ammonia removal by the flow stripping, but ozone oxidation predominates the removal reaction.
(3) The removal mechanism of ammonia nitrogen might be that ammonia nitrogen was mainly transformed into NO_3_^−^-N, less into NO_2_^−^-N, not into N_2_.

## Supplementary Files

Supplementary File 1
