# Supplementary material for: Treatment of Ammonia Nitrogen Wastewater in Low Concentration by Two-Stage Ozonization
Source: Int J Environ Res Public Health. 2015 Sep 23;12(9):11975–87. doi: 10.3390/ijerph120911975 (PMC4586718; doi:10.3390/ijerph120911975)
Supplement: Supplementary File 1 [file ijerph-12-11975-s001.pdf]

Treatment of Ammonia Nitrogen Wastewater in Low Concentration by Two-Stage Ozonization

Table S1. Ammonia nitrogen concentration of blank stripping over 3 L/min air stream.

Time (min)	Ammonia Nitrogen Concentration (mg/L)			
	Value 1	Value 2	Mean Value	SD
0	101.73	101.73	101.73	0
20	94.96	93.4	94.18	0.78
40	91.02	92.1	91.56	0.54
60	93.27	91.59	92.43	0.84
80	91.87	90.67	91.27	0.6
100	89.89	87.43	88.66	1.23
120	87.6	89.82	88.71	1.11

Table S2. The relationship between ozone concentration and flow rate.

Ozone Flow Rate (L/min)	Ozone Concentration (mg/L)			
	Value 1	Value 2	Mean Value	SD
0	0	0	0	0
0.3	24.98	26.68	25.83	0.85
0.5	29.87	30.61	30.24	0.37
0.8	48.88	53.12	51	2.12
1	57.73	58.55	58.14	0.41
1.3	72.34	71.3	71.82	0.52

Table S3. Effect of ozone flow rate on ammonia removal.

Time (min)	Removal Efficiency of Ammonia (%)			
	Value 1	Value 2	Mean Value	SD
0.3 L/min				
0	0	0	0	0
20	17.23	15.89	16.56	0.67
40	27.34	28.72	28.03	0.69
60	29.82	29.16	29.49	0.33
80	32.23	30.25	31.24	0.99
100	35.34	34.72	35.03	0.31
120	36.98	36.02	36.5	0.48
0.5 L/min				
0	0	0	0	0
20	21.75	22.79	22.27	0.52
40	31.26	30.04	30.65	0.61
60	32.78	33.94	33.36	0.58
80	37.23	36.51	36.87	0.36
100	38.24	38.2	38.22	0.02
120	39.47	38.05	38.76	0.71

Table S3. *Cont.*

Time (min)	Removal Efficiency of Ammonia (%)			
	**Value 1**	**Value 2**	**Mean Value**	**SD**
0.8 L/min				
0	0	0	0	0
20	16.8	18.3	17.55	0.75
40	29.35	27.65	28.5	0.85
60	36.98	35.88	36.43	0.55
80	38.95	39.79	39.37	0.42
100	40.27	40.23	40.25	0.02
120	39.81	41.27	40.54	0.73
1.0 L/min				
0	0	0	0	0
20	22.03	21.15	21.59	0.44
40	35.89	37.93	36.91	1.02
60	38.56	39.2	38.88	0.32
80	42.76	43.36	43.06	0.3
100	42.88	42.76	42.82	0.06
120	43.97	41.17	42.57	1.4
1.3 L/min				
0	0	0	0	0
20	17.64	19.06	18.35	0.71
40	24.35	23.65	24	0.35
60	35.78	36.76	36.27	0.49
80	37.17	37.73	37.45	0.28
100	37.69	37.81	37.75	0.06
120	38.24	38.44	38.34	0.1
1.5 L/min				
0	0	0	0	0
20	26.69	24.67	25.68	1.01
40	25.48	26.88	26.18	0.7
60	26.86	27.02	26.94	0.08
80	30.72	29.78	30.25	0.47
100	34.65	31.95	33.3	1.35
120	33.69	32.41	33.05	0.64

Table S4. Variation of pH *versus* reaction time at different ozone flow rates.

Time (min)	pH			
	Value 1	Value 2	Mean Value	SD
0.3 L/min				
0	10	10	10	0
20	9.6	9.4	9.5	0.1
40	9.3	9.3	9.3	0
60	9.2	9.2	9.2	0
80	9	9.2	9.1	0.1
100	8.8	9	8.9	0.1
120	8.5	8.3	8.4	0.1
0.5 L/min				
0	10	10	10	0
20	9.4	9.5	9.5	0.05
40	9.2	9.2	9.2	0
60	8.8	9	9	0.1
80	8.6	8.7	8.7	0.05
100	8.3	8.5	8.5	0.1
120	7	7.3	7.3	0.15
0.8 L/min				
0	10	10	10	0
20	9.4	9	9.2	0.2
40	8.9	8.7	8.8	0.1
60	7.6	7	7.3	0.3
80	7.4	6.8	7.1	0.3
100	6.7	6.5	6.6	0.1
120	6.5	6.1	6.3	0.2
1.0 L/min				
0	10	10	10	0
20	9.5	9.5	9.5	0
40	9.2	8.8	9	0.2
60	7.8	7.6	7.7	0.1
80	7.2	7	7.1	0.1
100	6.6	6.4	6.5	0.1
120	6	6	6	0
1.3 L/min				
0	10	10	10	0
20	9.5	9.3	9.4	0.1
40	8.7	8.5	8.6	0.1
60	7.6	7.4	7.5	0.1
80	7.2	7.4	7.3	0.1
100	6.6	7	6.8	0.2
120	6.3	6.5	6.4	0.1
1.5 L/min				
0	10	10	10	0
20	9.2	9	9.1	0.1
40	7.8	8	7.9	0.1
60	7.4	7.2	7.3	0.1
80	7.1	7.1	7.1	0
100	6.7	6.5	6.6	0.1
120	6.4	6.2	6.3	0.1

Table S5. Effect of initial pH on oxidation of ammonia.

Time (min)	Removal Efficiency of Ammonia (%)			
	Value 1	Value 2	Mean Value	SD
pH 8				
0	0	0	0	0
20	3.87	5.15	4.51	0.64
40	3.88	4.08	3.98	0.1
60	3.54	2.82	3.18	0.36
80	3.27	2.03	2.65	0.62
100	3.95	5.59	4.77	0.82
120	2.79	1.45	2.12	0.67
pH 9				
0	0	0	0	0
20	13.53	13.01	13.27	0.26
40	31.08	30.22	30.65	0.43
60	32.94	33.78	33.36	0.42
80	37.91	35.83	36.87	1.04
100	37.95	38.49	38.22	0.27
120	39.31	38.21	38.76	0.55
pH 10				
0	0	0	0	0
20	22.03	21.15	21.59	0.44
40	35.89	37.93	36.91	1.02
60	38.56	39.2	38.88	0.32
80	42.76	43.36	43.06	0.3
100	42.88	42.76	42.82	0.06
120	43.97	41.17	42.57	1.4
pH 11				
0	0	0	0	0
20	30.41	28.65	29.53	0.88
40	41.43	37.83	39.63	1.8
60	49.25	50.25	49.75	0.5
80	57.07	57.33	57.2	0.13
100	57.86	59.72	58.79	0.93
120	58.65	59.99	59.32	0.67
pH 12				
0	0	0	0	0
20	33.84	32.32	33.08	0.76
40	46.42	47.16	46.79	0.37
60	54.85	55.39	55.12	0.27
80	66.17	64.51	65.34	0.83
100	76.33	75.33	75.83	0.5
120	85.24	84.7	84.97	0.27

Table S6. Variation of pH *versus* reaction time at different initial pH.

Time (min)	pH			
	Value 1	Value 2	Mean Value	SD
		pH 8		
0	8	8	8	0
20	6.51	6.75	6.63	0.12
40	4.9	5.1	5	0.1
60	4.47	4.53	4.5	0.03
80	4.09	4.21	4.15	0.06
100	3.6	4	3.8	0.2
120	3.55	3.61	3.58	0.03
		pH 9		
0	9	9	9	0
20	8.01	8.09	8.05	0.04
40	6.3	7.3	6.8	0.5
60	6.1	6.5	6.3	0.2
80	4.9	4.7	4.8	0.1
100	4.34	4.26	4.3	0.04
120	4.02	3.98	4	0.02
		pH 10		
0	10	10	10	0
20	9.7	9.3	9.5	0.2
40	9.2	8.8	9	0.2
60	7.3	8.1	7.7	0.4
80	6.8	7.4	7.1	0.3
100	6.6	6.4	6.5	0.1
120	6.3	5.7	6	0.3
		pH 11		
0	11	11	11	0
20	9.8	9.6	9.7	0.1
40	9.5	9.3	9.4	0.1
60	8.8	9	8.9	0.1
80	7.5	7.66	7.58	0.08
100	7.21	6.59	6.9	0.31
120	6.75	6.51	6.63	0.12
		pH 12		
0	12	12	12	0
20	11.6	11.6	11.6	0
40	11.51	11.49	11.5	0.01
60	11.5	11.5	11.5	0
80	11.52	11.54	11.53	0.01
100	11.53	11.57	11.55	0.02
120	11.45	11.41	11.43	0.02

Table S7. The concentration changes of NH_4^+-N, TN, NO_3^--N and NO_2^--N with time at initial pH 10.

Time (min)	Concentrations at pH 10 (mg/L)			
	Value 1	Value 2	Mean Value	SD
		TN		
0	118.91	118.91	118.91	0
20	114.34	112.36	113.35	0.99
40	114.87	117.05	115.96	1.09
60	116.57	116.97	116.77	0.2
80	114.98	114.84	114.91	0.07
100	116.74	114.76	115.75	0.99
120	114.97	115.23	115.1	0.13
		NH_3-N		
0	122.19	122.19	122.19	0.00
20	96.35	95.27	95.81	0.54
40	75.84	78.34	77.09	1.25
60	68.29	75.07	71.68	3.39
80	69.20	69.94	69.57	0.37
100	69.95	69.79	69.87	0.08
120	71.88	68.46	70.17	1.71
		NO_2^--N		
0	0.12	0.14	0.13	0.01
20	0.08	0.06	0.07	0.01
40	0.15	0.19	0.17	0.02
60	0	0	0	0
80	0.13	0.13	0.13	0
100	0.25	0.29	0.27	0.02
120	0.16	0.18	0.17	0.01
		NO_3^--N		
0	0.05	0.03	0.04	0.01
20	19.67	19.55	19.61	0.06
40	37.28	36.98	37.13	0.15
60	45.49	44.51	45	0.49
80	42.12	44.24	43.18	1.06
100	45.37	44.57	44.97	0.4
120	43.71	43.19	43.45	0.26

Table S8. The concentration changes of NH_4^+-N, TN, NO_3^--N and NO_2^--N with time at initial pH 11.

Time (min)	Concentrations at pH 11 (mg/L)			
	Value 1	Value 2	Mean Value	SD
		TN		
0	112.07	112.07	112.07	0
20	98.24	100.82	99.53	1.29
40	101.14	104.1	102.62	1.48
60	100.24	99.9	100.07	0.17
80	102.87	104.19	103.53	0.66
100	103.02	102.58	102.8	0.22
120	106.78	104.64	105.71	1.07
		NH_3-N		
0	110.13	110.13	110.13	0.00
20	76.64	78.58	77.61	0.97
40	64.49	68.47	66.48	1.99
60	55.89	54.79	55.34	0.55
80	47.29	46.99	47.14	0.15
100	46.40	44.36	45.38	1.02
120	45.54	44.06	44.80	0.74
		NO_2^--N		
0	0.1	0.1	0.1	0
20	0.14	0.12	0.13	0.01
40	0.14	0.12	0.13	0.01
60	0.12	0.14	0.13	0.01
80	0.08	0.12	0.1	0.02
100	0	0	0	0
120	0.25	0.29	0.27	0.02
		NO_3^--N		
0	0.43	0.43	0.43	0
20	22.49	20.93	21.71	0.78
40	37.76	37.24	37.5	0.26
60	44.51	41.91	43.21	1.3
80	55.6	54.22	54.91	0.69
100	56.35	55.01	55.68	0.67
120	60.22	58.98	59.6	0.62

Table S9. The removal of ammonia by two stages of ozone oxidation.

**Time (min)**	**Concentration of ammonia nitrogen (mg/L)**			
	**Value 1**	**Value 2**	**Mean value**	**SD**
0	30.54	30.54	30.54	0
20	22.01	23.95	22.98	0.97
40	12.87	15.09	13.98	1.11
60	11.85	12.03	11.94	0.09
80	11.33	10.81	11.07	0.26
100	11.23	10.91	11.07	0.16
120	11.41	11.31	11.36	0.05

© 2015 by the authors; licensee MDPI, Basel, Switzerland. This article is an open access article distributed under the terms and conditions of the Creative Commons Attribution license (http://creativecommons.org/licenses/by/4.0/).
